# Effect of Bhramari Pranayama on the Acoustic and Aerodynamic Parameters of Voice in Normophonic Females

**DOI:** 10.1155/2018/4176803

**Published:** 2018-08-06

**Authors:** Usha Manjunatha, Jayashree S. Bhat, Kumar B. Radish, Gagan Bajaj, Poovitha Shruthi, Priyanka Suresh Nayak, Saniya Mariam Rasheeka

**Affiliations:** ^1^Department of Audiology and Speech Language Pathology, Kasturba Medical College, Mangalore, Manipal Academy of Higher Education, Manipal, India; ^2^Centre for Integrative Medicine and Research (Yoga), Manipal Academy of Higher Education, Manipal, India

## Abstract

**Summary. Objective:**

Pranayama is known for improving various health conditions. The present study is aimed at investigating the effects of bhramari pranayama on aerodynamic and acoustic parameters of voice in healthy individuals.

**Study Design:**

This is a pretest-posttest design study.

**Methods:**

A total of 24 participants in the age range of 20 to 25 years completed the bhramari pranayama regimen for 30 sessions. Aerodynamic and acoustic assessments were done before and after pranayama sessions. Maximum phonation duration, pitch, loudness, subglottal pressure, glottal airflow, laryngeal resistance and conductance along with acoustical parameters such as average F0, jitter, and shimmer, soft phonation index, noise to harmonic ratio, cepstral peak prominence, and smoothened cepstral peak prominence were analysed.

**Results:**

The results revealed significant improvement in the maximum phonation duration, glottal airflow and pressure, average fundamental frequency, and cepstral peak prominence after practice suggesting that it has an effect on voice parameters.

**Conclusion:**

Bhramari pranayama is effective in improving the acoustic and aerodynamic parameters of voice. The same needs to be investigated in hyper- and hypofunctional voice disorders in the future studies.

## 1. Introduction

India, being the place of birth for many ancient traditional cultures, has given traditional form of postures called asanas in yoga and breathing techniques called pranayama, now being practiced worldwide. Yoga has got many names, forms, styles, and benefits. It is practiced and believed to be essential to achieve the sense of both mental and physical wellbeing; it is also considered as a means for energizing and strengthening the physical body. Pranayama, being one of the eight limbs of yoga, deals with the correct breathing pattern and many exercises related to breath [1–4]. It regulates the vital forces of the body and helps in curing lung related and voice related disorders [5–9].

There are various styles and forms of pranayama to achieve good breathing pattern, to cure many disorders. One such technique called “bhramari” is intensely practiced in yogic exercises. The bhramari pranayama is a slow breathing technique and can be practiced irrespective of the age and gender [10]. The bhramari pranayama is a Sanskrit term, in which “bhramari” means a black bumble bee, and “pranayama” (prana + yama) means control (yama) of vital energy (prana), the subtlest form of universal and breathing energy [11]. Bhramari pranayama is said to activate one of the energy focal centre called “Vishuddhi chakra” located in the throat region, which is responsible for activating the voice box used for speech communication [12].

Bhramari pranayama technique includes making a humming sound like a bee during exhaling [4, 8, 10, 13, 14], in a relaxed upright sitting posture. The eyes must be closed during this process to cut off external inputs of sound and sight to internalize the consciousness. The sensation of vibration on the facial region is experienced by placing the fingers on the different regions of the face. Yogic practitioners follow different styles and methods of performing these exercises, and the duration is mentioned as at least ten minutes. Initially, a single cycle of bhramari is performed and, with practice, duration can be increased to ten minutes [15].

In literature, many benefits of practicing bhramari pranayama have been reported. Few of them are reducing the stress, anxiety, anger, and frustration; reducing the blood pressure; providing a good sonorous voice; and removing throat ailments [4, 14]. There are studies reporting the scientific reasons behind the practice of this yoga, pranayama, and its attributes in improving the health condition of many pathological cases such as asthma and other respiratory disorders [5]. Moore (2012), who is a speech language pathologist by profession and also a yoga teacher, has reported the positive outcomes of modified yoga technique practice in adjunct with voice therapy on patients with muscle tension dysphonia. Hutton, Rogers, and Doan in 2014 have stated that regular practicing of few asanas (body postures), Mudras (hand postures), and meditation helps in improving the body health, voice, and performance anxiety in singing teachers. They have documented few postures like basic chest opening and spine-lengthening and some breathing techniques which help the singing tutors to hold the breath for longer duration and sing long phrases without much effort. They also report keeping the blood pressure in normal range by practicing yoga and pranayama. Rao, Hongsandra, and Nagendra in 2014 documented the role of few postures and breathing exercises in yoga and pranayama in correcting the functional voice disorders. They also have reported similar asanas and pranayama techniques as reported by Hutton et al. which help in increasing the lung capacity, reducing the tension in vocal muscles, and in turn improving the voice quality. However, these findings were not experimentally investigated.

### 1.1. Need for the Study

Voicing is a complex dynamic product of the coordination of the various subsystems which includes respiratory, phonatory, resonatory, articulatory, and nervous system. Earlier studies have reported benefits of pranayama on general heath, specifically in disorders like asthma, anxiety, depression, and hypertension [10], with few addressing the effect on voice based on the breathing techniques mentioned in pranayama. Bhramari pranayama has been reported in the traditional yogic literature to improve the conditions of throat alignments, thereby improving voice. However, there is no experimental evidence to support this assumption. Also there is a need for theoretical framework for the physiological functions of bhramari pranayama on voice. Since pranayama deals with breathing and breathing is the source of voice production, it is hypothesized that bhramari pranayama will have its implications for improving voice of affected individuals. However, the same cannot be investigated in individuals with voice disorders due to the lack of experimental evidence in the literature. Hence the present study investigated the effect of bhramari pranayama on the acoustic and aerodynamic parameters of voice in healthy normal females.

## 2. Method

### 2.1. Design and Participants

The pretest-posttest study design which got approved from institutional ethical committee included 26 females within the age range of 20 to 25 years (mean age: 20.87 ± 1.15 years). With the help of a questionnaire, participation eligibility was ascertained. All females were non-yoga practitioners and did not have formal training in yoga and pranayama. Two of the 26 participants discontinued the study as they could not complete the specified pranayama regimen due to personal reasons. A written informed consent was obtained prior to the recruitment of the participants. All the participants underwent hearing screening to ascertain normal hearing sensitivity. Information regarding their menstrual cycle was collected to ensure that they were falling in 5th to 15th day of the cycle; females with the history of surgery of the vocal apparatus, vocal pathology, respiratory tract infections, asthma, smoking, and consuming oral contraceptives were excluded from the study.

### 2.2. Instrumentation

- Acoustical recording was performed using Computerized Speech Lab (KayPENTAX 4150) system. For analysis Multidimensional Voice Program (MDVP), a module of CSL, was used. The system uses sustained phonation samples for analysis and the extraction of acoustic parameters.
- For the same acoustic samples, cepstral analysis was carried out using z-tool, also called speech tool program software which uses Hillenbrand algorithm [16].
- Aerodynamic recording and analysis were carried out using a calibrated Phonatory Aeroview System (version 1.6.3 by Glottal Enterprises). This system consists of an oronasal mask with the handle, wherein pressure and airflow transducers are connected. A tube from pressure transducer sits in the oral cavity to measure the oral pressure during the data recording; simultaneously airflow during the sound production is picked by the airflow transducer.

### 2.3. Intervention

A speech language pathologist by profession and also a certified yoga practitioner conducted the pranayama sessions for all the participants. Participants were called for the session after a gap of minimum 3 hours of their food intake [17]. Session began with raising the awareness of the proper breathing pattern where all the participants were asked to place their hand on abdomen and correct their breathing pattern by contracting the abdomen during exhalation and releasing the abdomen during inhalation. Also the posture orientation was given and they were instructed to sit on a chair in upright position by keeping the neck straight so that the position of the plumb line aligned with the external ear, midway through the shoulders with straight back [18]. The upper body was perpendicular to the thighs and knees bent at right angle and foot flat and grounded. Individuals were supposed to evenly distribute the whole body weight on both the hips [19]. Arms were rested on the thighs or the arm rest of the chair, so as to keep the upper body calm and relaxed, not rigid, and to keep the shoulders low and the neck muscles without any strain [20]. For proper understanding of breathing pattern, initially the ratio of inhalation and exhalation was taken as 1:1 since all the participants were new to the concept of yoga and pranayama. Once all the participants attained correct breathing pattern and posture, bhramari pranayama [10] was taught to them by maintaining the posture, to produce a resonating sound like a mumble bee while exhaling through the nostrils by keeping the oral cavity closed. During the process, participants were asked to sense the vibration in the facial region due to the vocalization. Participants were divided into 2 groups for better monitoring, and home practice was not advised. Each session consisted of performing the bhramari pranayama for 12 cycles, and total of 30 sessions were completed in a month.

### 2.4. Procedure

Recordings for the acoustic and aerodynamic measurements were done in a quiet room in the voice lab. Voice samples were collected using a dynamic microphone into Computerized Speech Lab system. Participants were seated on chairs comfortably and asked to produce sustained phonation of /a/, /i/, and /u/ at their comfortable pitch and loudness. Microphone to mouth distance was kept approximately constant as 15 cm. Three trials were recorded and every second trial was considered for the analysis to eliminate the mistakes in first trial and vocal fatigue in the last trial.

Aerodynamic measurements were taken using Phonatory Aeroview System. System was calibrated using PC-H1 pressure calibrator and FC-C flow calibrator before the data recording. Participants were asked to hold the oronasal mask and achieve the seal before the sound production. All of them were instructed to produce alternating syllable string /pa/ in the mask provided. Pre-recording was done in the 1st session of the pranayama regimen and after assessment was carried out for all the participants for both acoustic and aerodynamic parameters after the 30th session using the similar tasks in baseline assessments.

### 2.5. Acoustic and Aerodynamic Analysis

Medial stable 10 seconds of sustained vowel samples of /a/, to avoid fade-in and fade-out effects of signal, were taken for acoustical analysis in Multidimensional Voice Profile (MDVP) and z-tool for measuring parameters such as the following:

- average F0: the average mean of vocal fold vibration across the length of the recorded voice sample of an individual;
- jitter and shimmer: the fluctuation rate or vocal fold vibratory characteristics in terms of frequency and intensity [21];
- noise to harmonic ratio (NHR): the information on presence of noise in a particular voice signal compared to harmonic organization [22, 23];
- soft phonation index (SPI): a parameter to check for noise or breathiness in the voice signal; high rate of SPI indicates poor adduction of vocal folds and more breathy voice [23];
- cepstral peak prominence and smoothened cepstral peak prominence (CPP and sCPP): the information on harmonic structure in the voice signal; they are the best predictors of dysphonic components of voice compared to NHR or SPI [16, 24, 25].

**Figure 1:**
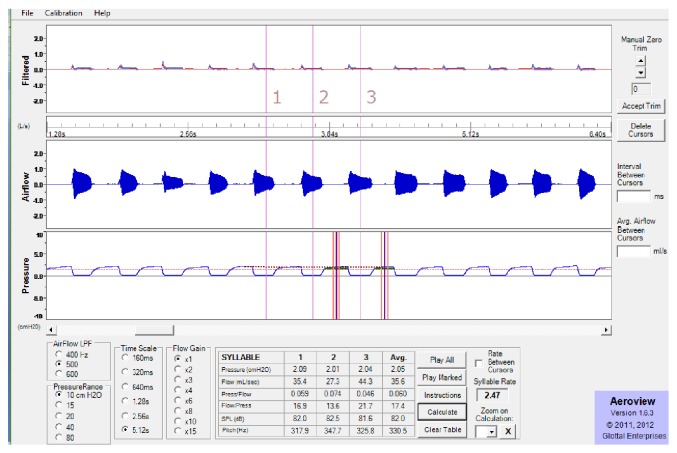
Phonatory Aeroview System analysis window showing the selection of three consecutive syllables of /pa/.

For aerodynamic analysis, consecutive three syllables of /pa/ were selected and the average values of the following parameters were calculated by the system algorithm along with pitch and sound pressure level in Aeroview analysis software (Figure 1):

- subglottal pressure: average pressure at the level or below the glottis;
- glottal flow: mean airflow at the level of glottis;
- glottal resistance: the resistance offered by the glottis at the time of sound production obtained by calculating pressure by airflow;
- glottal conductance: the allowance of air through the glottis at the time of sound production obtained by calculating airflow by pressure.

### 2.6. Statistical Analysis

The statistical software EZR (Easy R), 64 bits, for windows was used for the data analysis. Mean, median, and standard deviation were obtained for entire data. For the data which did not follow normal distribution by Shapiro–Wilk normality distribution test, Wilcoxon signed rank test was conducted, and for the data which followed normal distribution, paired* t*-test was conducted. To see the significant difference between the groups, the level of significance of p-value was kept at 0.05.

## 3. Results

### 3.1. Aerodynamic Measures

Table 1 shows the mean and standard deviation for maximum phonation duration (MPD) of the sustained vowels /a/, /i/, and /u/ for all the 24 participants before and after practicing bhramari pranayama for 30 sessions along with paired* t*-test results, since Shapiro–Wilk test showed that the data were normally distributed. Mean of MPD for all the vowels increased after practicing bhramari pranayama. Also there was a significant difference between results before and after practice for /a/, /i/, and /u/ of paired* t*-test.

Table 2 shows the median, interquartile range, and Wilcoxon signed rank test for the aerodynamic parameters measured in Aeroview software which did not follow normal distribution.  Table 3 shows mean, standard deviation, and paired* t*-test results of pitch and SPL which followed normal distribution. As tabulated below, there was increase in the mean scores of glottal flow, conductance, pitch, and sound pressure level, but inferential statistics revealed significant difference only in glottal flow, pitch, and SPL during the production of the pressure consonant /pa/. Similarly there was decrease in mean scores of subglottal pressure and glottal resistance, but significant difference was observed only in subglottal pressure before and after bhramari pranayama sessions.

### 3.2. Acoustical Analysis

Table 4 shows mean, standard deviation, and paired* t*-test results for all the acoustic parameters except for jitter and soft phonation index which followed normal distribution. Median, interquartile range, and Wilcoxon signed rank test for jitter and SPI are tabulated in Table 5. There was increase in the mean score of average F0, CPP, and sCPP after practicing bhramari pranayama but significant difference was shown only in Avg F0 (p = 0.022) and CPP (p = 0.0015). Mean scores of jitter and shimmer decreased after practice of bhramari pranayama, but inferential statistics showed no significant difference.

## 4. Discussion

The main objective of the current study was to investigate the effects of bhramari pranayama practice on the aerodynamic and acoustical measures of voice in healthy adult females. The results indicated significant improvements in the maximum phonation duration for all the sustained vowels. This indicates that the practice of bhramari pranayama improved the respiratory phonatory coordination in the selected females and also that with extensive practice one can achieve breath control. Regular practice of pranayama including bhramari pranayama is reported to be efficient in improving the lung functions in pathological cases like traumatic spinal cord injury [8, 9, 26–28].

In normal breathing, individual generally neglects the posture and the breathing pattern. During the practice of bhramari pranayama, one should give concentration towards the posture and breathing pattern. Hence the inspired air can be uniformly distributed in the lungs and can occupy more volume, which makes the person able to inspire deeply and exhale the same amount of air without causing any strain [29]. This shows that bhramari pranayama along with postural management has an effect on increasing the lung functions and breathing pattern, which justifies the results of the present study.

The results of aerodynamic analysis revealed significant improvements in subglottal pressure, glottal airflow, pitch, and loudness after bhramari pranayama practice. All these parameters such as glottal pressure, flow, resistance, and conductance are interrelated and cannot be explained individually. The improvement that is seen in the lung functioning (MPD) and the loudness level can be attributed to increased glottal airflow [30]. Also, it was observed that even though the subglottal pressure was decreased, there was an increase in the sound pressure level due to increase in the airflow. These findings are in agreement with study done in 1964, where it is concluded that vocal intensity can change even though the flow rate increases or remains the same [30]. Pitch and loudness are directly related and only trained singers can control either of the parameters during the production of different registers [31]; otherwise, intensity goes high when they produce high pitch; also increase in the glottal flow results in increase in frequency and sound pressure level [32]. In the current study both pitch and SPL have shown significant difference after the practice of bhramari pranayama.

Researchers have shown significant increase in subglottal pressure, maximum flow declination rate, and translaryngeal airflow in maximum effortful speech when compared to normal speech. This makes the perilaryngeal area more strained and results in hyperadduction of vocal folds [33]. The results of the current study revealed decreased mean scores of subglottal pressure and laryngeal resistance after the practice sessions of bhramari pranayama. This can be attributed to the reductions in the level of vocal effort during the voice production after the practice. Although there was no significant difference in glottal resistance and conductance, there was a difference in the mean scores but greater significance is observed in subglottal pressure and airflow which can be attributed to the positive effects of bhramari pranayama by reducing the vocal effort and phonation threshold pressure.

There are many studies on bhramari pranayama with respect to health, cardiovascular disease, breathing related problems, etc., but no studies to support the effect of bhramari pranayama practice on changing the voice quality. The production of vibrating sound during the practice of this pranayama resonates the entire vocal tract by bringing the forward focus and gives the tactile vibratory sensation to the practitioner. This converts the entire aerodynamic energy into an acoustical form with more strength and ringing sensation in the voice. Also this practice brings out the easy onset of phonation when practiced with good posture and relaxed manner. Even though there was no significant difference seen in all the acoustical parameters, mean scores of average fundamental frequency and CPP showed significant improvement after the practice sessions of bhramari pranayama. This indicates improved harmonic organization after bhramari pranayama practice. Cepstrum parameters are considered as the predictors of dysphonia or breathy voice [34]. Increased CPP after bhramari pranayama practice indicates better or improved harmonic organization in the voice sample [16, 33, 35, 36] and the current study results are in agreement with the available literature. Increase in F0 and CPP indicates the more flexibility in voicing [33] which is achieved by practicing bhramari pranayama. Since voicing plays a key role in communication, it is important to maintain the same quality and this applies especially to professional voice users. Bhramari pranayama as a healthy regimen can be considered as one of the vocal improvement exercises in voice rehabilitation in healthy adults. As explained in Resonant Voice Therapy [35], bhramari pranayama is also concentrating on forward focusing of voice with minimum vocal effort, thereby producing the good quality and strongest voice possible. The barely adducted vocal folds during bhramari pranayama are effective in reducing the phonation threshold pressure and make the easy onset of phonation.

Limitation of this study is considering only female participants. Since this is the primitive research of bhramari pranayama on voice, to maintain the homogeneity only one gender was considered. Future studies can be done on various age groups and genders.

## 5. Conclusion

The present study investigated the effect of bhramari pranayama on the acoustic and aerodynamic parameters of voice in healthy normal individuals. The results revealed significant improvement in the maximum phonation duration, glottal airflow and pressure, average fundamental frequency, and cepstral peak prominence after practice sessions of bhramari pranayama suggesting that bhramari pranayama has an effect on voice related acoustic and aerodynamic parameters. The result of the present study is an initial step towards creating evidence base for the bhramari pranayama towards voice improvement in healthy adults. However, the same needs to be investigated in the disordered voice population. Further studies should address this issue in hyper- and hypofunctional voice disorders.

## Figures and Tables

**Table 1:** Mean, standard deviation, and Paired *t*-test of maximum phonation duration (MPD) for all the participants before and after bhramari pranayama.

	**Mean**		**SD**		
	**Pre**	**Post**	**Pre**	**Post**	**P**
**/a/ (Secs)**	14.80	18.15	3.25	3.46	< 0.001*∗*
**/i/ (Secs)**	19.12	21.86	5.74	5.82	< 0.001*∗*
**/u/ (Secs)**	18.71	21.74	5.01	6.33	< 0.001*∗*

**Table 2:** Median, interquartile range, and Wilcoxon sign rank test of aerodynamic measures for all the participants before and after bhramari pranayama.

	**Median**		**IQ range **		
	**Pre**	**Post**	**Pre**	**Post**	**P**
**Pressure (cmH2O)**	8.00	5.54	7.12-8.76	5.73-5.54	0.0366*∗*
**Flow (L/sec)**	0.11	0.13	0.11-0.25	0.13-0.40	0.0291*∗*
**Pressure/Flow**	0.17	0.18	0.17-0.69	0.18-0.69	0.8996
**Flow/Pressure**	1.46	1.55	1.46-6.05	1.55-6.00	0.5457

**Table 3:** Mean, standard deviation, and paired *t*-test of pitch and SPL for all the participants before and after bhramari pranayama.

	**Mean**		**SD**		
	**Pre**	**Post**	**Pre**	**Post**	**p**
**Pitch (Hz)**	223.01	253.25	46.30	58.90	0.008*∗*
**SPL (dB)**	75.87	81.93	6.35	4.47	0.0004*∗*

**Table 4:** Mean, standard deviation, and paired *t*-test of acoustical parameters for all the participants before and after bhramari pranayama.

	**Mean**		**SD**		
	**Pre**	**Post**	**Pre**	**Post**	**P**
**Avg F0 (Hz)**	211.27	217.32	47.49	50.33	0.018*∗*
**Shimmer (**%**)**	2.98	2.74	0.84	0.67	0.155
**NHR**	0.11	0.11	0.01	0.01	0.894
**CPP (dB)**	18.28	20.77	1.81	4.49	0.003*∗*
**sCPP (dB)**	8.78	9.41	1.30	2.14	0.092

**Table 5:** Median, interquartile range, and Wilcoxon sign rank test of Jitter and SPI for all the participants before and after bhramari pranayama.

	**Median**		**IQ Range**		
	**Pre**	**Post**	**Pre**	**Post**	**p**
**Jitter (**%**)**	0.44	0.46	0.75-0.88	0.58-0.79	0.6431
**SPI**	8.20	8.46	12.03-15.88	11.09-13.29	0.2643

## Data Availability

The data used to support the findings of this study are available from the corresponding author upon request.
